# Binding specificity and function of the SWI/SNF subunit SMARCA4 bromodomain interaction with acetylated histone H3K14

**DOI:** 10.1016/j.jbc.2021.101145

**Published:** 2021-08-30

**Authors:** Paul Enríquez, Krzysztof Krajewski, Brian D. Strahl, Scott B. Rothbart, Robert H. Dowen, Robert B. Rose

**Affiliations:** 1 Department of Molecular and Structural Biochemistry, North Carolina State University, Raleigh, North Carolina, USA; 2 Department of Biochemistry and Biophysics, The University of North Carolina at Chapel Hill, Chapel Hill, North Carolina, USA; 3 Department of Epigenetics, Van Andel Institute, Grand Rapids, Michigan, USA; 4 Integrative Program for Biological and Genome Sciences, The University of North Carolina at Chapel Hill, Chapel Hill, North Carolina, USA; 5 Department of Cell Biology and Physiology, The University of North Carolina at Chapel Hill, Chapel Hill, North Carolina, USA; 6 Department of Biology, The University of North Carolina at Chapel Hill, Chapel Hill, North Carolina, USA

**Keywords:** epigenetics, protein structure, CRISPR/Cas, *C.elegans*, bromodomain, protein microarray, H3K14ac, SWI/SNF, SMARCA4, BRG1, BD, bromodomain, H3K14ac, histone H3 sequence with K14 acetylated

## Abstract

Bromodomains (BD) are conserved reader modules that bind acetylated lysine residues on histones. Although much has been learned regarding the *in vitro* properties of these domains, less is known about their function within chromatin complexes. SWI/SNF chromatin-remodeling complexes modulate transcription and contribute to DNA damage repair. Mutations in SWI/SNF subunits have been implicated in many cancers. Here we demonstrate that the BD of *Caenorhabditis elegans* SMARCA4/BRG1, a core SWI/SNF subunit, recognizes acetylated lysine 14 of histone H3 (H3K14ac), similar to its *Homo sapiens* ortholog. We identify the interactions of SMARCA4 with the acetylated histone peptide from a 1.29 Å-resolution crystal structure of the *Ce*SMARCA4 BD–H3K14ac complex. Significantly, most of the SMARCA4 BD residues in contact with the histone peptide are conserved with other proteins containing family VIII bromodomains. Based on the premise that binding specificity is conserved among bromodomain orthologs, we propose that loop residues outside of the binding pocket position contact residues to recognize the H3K14ac sequence. CRISPR-Cas9-mediated mutations in the SMARCA4 BD that abolish H3K14ac binding *in vitro* had little or no effect on *C. elegans* viability or physiological function *in vivo*. However, combining SMARCA4 BD mutations with knockdown of the SWI/SNF accessory subunit PBRM-1 resulted in severe developmental defects in animals. In conclusion, we demonstrated an essential function for the SWI/SNF bromodomain *in vivo* and detected potential redundancy in epigenetic readers in regulating chromatin remodeling. These findings have implications for the development of small-molecule BD inhibitors to treat cancers and other diseases.

Bromodomains (BD) are highly conserved epigenetic reader modules that recognize acetyl-lysine (Kac) on histones and other proteins (1, 2). (Note: for clarity, histone residues will be referred to in one-letter code, and BD residues will be referred to in three-letter code.) In the nearly 30 years since BDs were first identified, the chromatin field has accumulated a wealth of biophysical, structural, and biochemical data on BDs and other epigenetic readers (3, 4). Structures from eight human BD families have been solved (1), and small molecules are now available for BD inhibition (5). Most BDs and their binding partners have been well characterized *in vitro* and in various cell lines. The precise functional role and mechanistic underpinnings of BD–histone target specificity at the organismal level, however, remain largely unknown. To date, only a handful of studies have examined BDs *in vivo* and have done so only in the context of chemical-probe inhibition (6, 7, 8, 9), which has been challenging due to issues related to lack of specificity, acquired resistance, off-target effects, and cytotoxicity (10, 11, 12, 13). Despite the advent of genome editing, no research group has—to our knowledge — disrupted BD–histone interactions in a complex multicellular organism to investigate the contributions of BD binding on cell differentiation and development. Nor have researchers begun to mine the vast structural and sequence data already available to elucidate potential global patterns of specificity and plasticity common among BD subfamilies targeting the same marks. This paper addresses these gaps in studies of chromatin regulation by epigenetic readers.

SMARCA4/BRG1 is an essential catalytic core subunit of the roughly two-megadalton Switch/Sucrose Nonfermenting (SWI/SNF) multiprotein complex, which uses the energy of ATP hydrolysis to remodel chromatin by perturbing interactions between histone core particles and DNA (14, 15). SMARCA4 can remodel nucleosomal substrates by itself *in vitro* (16, 17) and is functionally and structurally conserved among eukaryotes (14, 18). This remodeling enzyme possesses intrinsic ATPase and helicase activity and has a C-terminal BD motif capable of recognizing lysine 14 acetylation on histone H3 (H3K14ac) (19) (Fig. 1*A*). Malfunction or loss of the SMARCA4 subunit has been implicated in numerous cancers, aberrant patterns of cell differentiation, inflammatory responses, and metabolic dysfunction (20, 21, 22, 23, 24).

**Figure 1:**
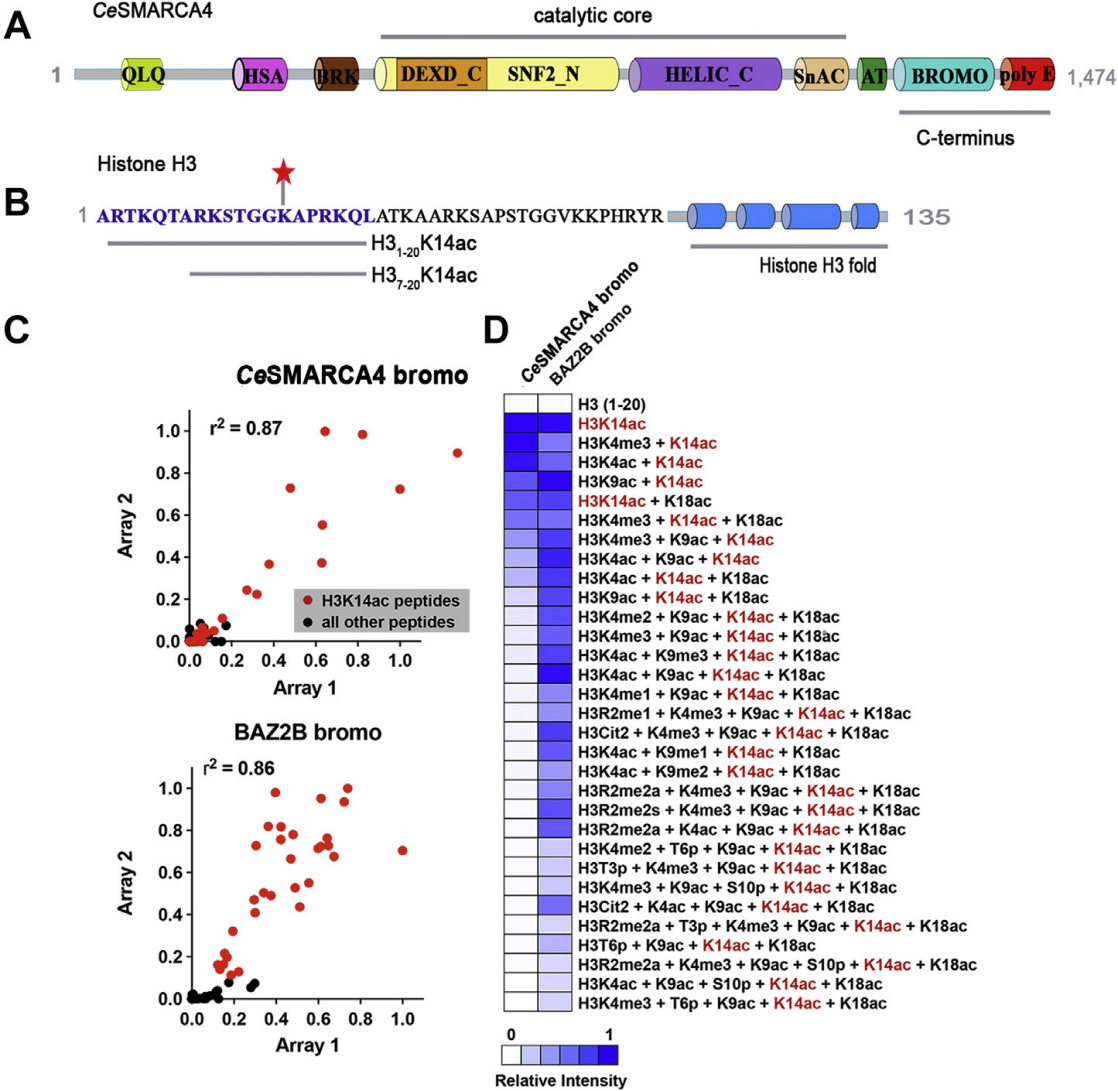
**Histone peptide interactions between *Ce*SMARCA4 bromodomain and histone H3 modified peptides.***A*, domain architecture of full-length *Ce*SMARCA4 protein. QLQ, Gln-Leu-Gln domain; HSA, Helicase/SANT-associated domain; BRK, Brahma and Kismet domain; DEXD_C, DEAD-like helicase superfamily domain; SNF2_N, SNF2 family N-terminal domain; HELIC_C, Helicase superfamily C-terminal domain; SnAC, Snf2 ATP Coupling; AT, tandem AT hooks; Bromo, Bromodomain; polyE, C-terminal polyglutamate region. *B*, amino acid sequence of the N-terminus of histone H3. The sequence of the intrinsically disordered residues of the H3 N-terminal tail is shown in text, followed by a *cartoon* illustration of the histone H3 globular domain comprised of four α-helices. The sequences of the H3 peptides used for ITC and crystallization are shown in blue text. Acetylation of lysine 14 (K14ac) is shown as a *red star*. *C*, correlation of binding data between two replicate arrays hybridized with recombinant *Ce*SMARCA4 or BAZ2B bromodomains. *D*, heatmap of normalized mean signal intensities (*white*, no binding; *blue*, strong binding) for all H3K14ac-containing peptides bound with *Ce*SMARCA4 BD or BAZ2B BD. Arrays consist of a library of approximately 350 unique histone peptides and are printed in horizontal triplicates in two disparate locations on each array.

How SMARCA4 targets, is recruited to, and interacts with chromatin substrates to induce ATP-dependent nucleosome remodeling has been a long-standing question. Recent structures of the SMARCA4 catalytic-core domain in complex with a nucleosome core particle, as well as a nucleosome-bound SWI/SNF complex comprising SMARCA4 and nine auxiliary subunits, provided insights into the mechanism of SWI/SNF remodeling (25, 26). But the structural basis underlying SMARCA4 recognition of its histone tail target has remained elusive despite the existence of SMARCA4 structures in the apo state (1, 27, 28) and in complex with a chemical probe (PFI-3) selective for four family VIII BD (29). More recently, a structure of the *Saccharomyces cerevisiae* Sth1 (*Sc*Sth1) BD in complex with H3K14ac (30) revealed the histone binding mode for the catalytic core of the yeast RSC (Remodel the Structure of Chromatin) complex—a paralog that is at least ten times more abundant than SWI/SNF and differs in both component organization and physiological function (31, 32). Unlike SMARCA4, which exclusively targets mono- and di-acetylated H3 tails, Sth1 binds more promiscuously to H3K14ac and other mono-acetylated lysine posttranslational modifications (PTMs) on histones H3 and H4, including H4K20ac, H3K18ac, and H3K27ac (30). Thus, a more complete understanding of the specific contributions of the SMARCA4 BD to SWI/SNF biology requires structural and, more importantly, *in vivo* validation in a complex multicellular organism, which is sorely lacking in the field.

Here, we report findings from structural, physicochemical, and *in vivo* genetic investigations of the SMARCA4 epigenetic-reader domain. We use the reference nematode *Caenorhabditis elegans* and its SMARCA4 ortholog (SWSN-4 or *Ce*SMARCA4)—which is conserved among eukaryotes and constitutes the only SWI/SNF ATPase in the worm—as a model. Using peptide microarrays, we show that the *Ce*SMARCA4 BD is, like its human ortholog, highly selective for H3K14ac. We analyzed the basis for H3K14ac-binding selectivity by solving a 1.29 Å resolution structure of the *Ce*SMARCA4 BD–H3K14ac complex and comparing it to other BDs that bind the mark. Based on the premise that binding specificity is evolutionarily conserved within each BD ortholog, we identify residues distant from the H3 binding site that contribute to selective H3K14ac recognition, which could be exploited to create highly specific, next-generation BD chemical probes. To examine the functional significance of the *Ce*SMARCA4 BD–H3K14ac interaction *in vivo*, we engineered specific BD mutations into the *C. elegans swsn-4* gene using CRISPR-Cas9 genome editing (33). While BD mutations that abolish acetyl-lysine binding *in vitro* only modestly impact *C. elegans* viability, we found that a combination of SMARCA4 BD binding mutants with genetic inactivation of the *pbrm-1* gene, which encodes an accessory SWI/SNF subunit, resulted in enhanced embryonic lethality and fertility defects. These data suggest that the SMARCA4 BD plays a significant and redundant role with other members of the SWI/SNF complex *in vivo*. Collectively, our findings underscore a pressing need for *in vivo* validation of studies employing BD inhibitors and *in vitro*-derived data to interpret the functional roles of epigenetic readers in chromatin regulation and signaling.

## Results

### The *C. elegans* SMARCA4-BD binds selectively to H3K14ac *via* its bromodomain with low micromolar (μM) affinity

To assess whether SMARCA4 BD–H3K14 binding is conserved between mammals and *C. elegans*, we screened a recombinantly expressed and purified Glutathione S–Transferase (GST)–bromodomain fusion protein against a microarrayed library of 300+ biotinylated histone peptides (34). The library comprises peptides from all core histones, as well as the major histone variants, in single and combinatorial modification states. Each peptide contains a terminal biotin tag for immobilization on streptavidin-coated glass slides. The *Ce*SMARCA4 GST–BD fusion bound specifically to H3K14ac-containing peptides. Co-occurrence of H3K9ac or H3K18ac weakened the interaction, and *Ce*SMARCA4 did not bind to unmodified histone H3 N-terminal tails (Fig. 1, *B*–*D*). Overall, the *Ce*SMARCA4 BD is highly selective for mono-acetylated H3K14 tails, suggesting functional and structural conservation between the human and worm proteins. We also tested the human BAZ2B BD, a family V BD, and found that, like the *Ce*SMARCA4 (family VIII) BD, it is highly selective for H3K14ac-modified peptides; however, BAZ2B also recognizes poly-acetylated tails, binding to di- (H3K9acK14ac) and tri-acetylated (H3K9acK14acK18ac) peptides (Fig. 1*D*) (35, 36, 37).

**Figure 2:**
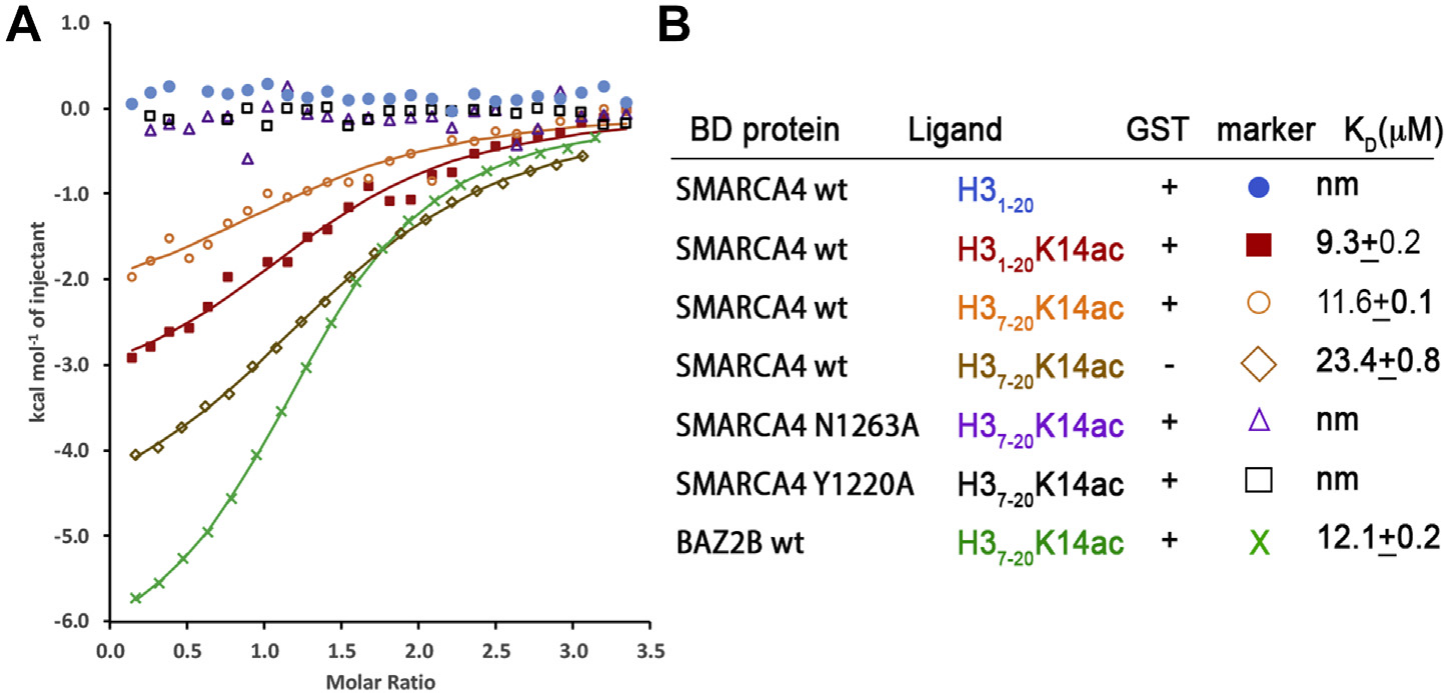
**Binding affinity of *Ce*SMARCA4 bromodomain for histone H3 peptides.***A*, isothermal titration calorimetry binding curves and *B*, the corresponding K_d_s of histone H3 N-terminal oligopeptides against the *Ce*SMARCA4 bromodomain. ITC titration curves of GST-*Ce*SMARCA4 fusion proteins bound to unmodified H3_1–20_, H3_1–20_K14ac, and H3_7–20_K14ac histone oligopeptides (*blue*, *red*, *orange*, respectively). Removal of the GST tag did not alter binding affinity (*brown diamond*). *Ce*SMARCA4 mutations Tyr1220Ala (*black squares*) and Asn1263Ala (*purple triangles*) abolished K14ac binding. BAZ2B BD binding to H3_7–20_K14ac (*green*).

We next sought to quantitate the *Ce*SMARCA4 GST–BD binding affinity for H3_1–20_K14ac and H3_7–20_K14ac histone peptides (Fig. 2*A*) in solution *via* isothermal titration calorimetry (ITC). We calculated single-digit and low double-digit μM dissociation constants—*K*_*D*_ = 9.3 ± 0.2 and 11.6 ± 0.1 μM, respectively—for these modified histone peptides (Fig. 2*B*). To further assess whether GST interferes with binding affinity of the complex, we cleaved off the GST tag and retested binding for H3_7–20_K14ac *via* ITC, which yielded *K*_*D*_ = 23.4 ± 0.8 μM and confirmed that GST does not interfere with BD binding. Our results contrast sharply with previous studies of the binding affinities between the *Homo sapiens* SMARCA4/2 (*Hs*SMARCA4/2) ortholog and titrated H3K14ac-modified histone peptides *via* NMR perturbation experiments, which reported *K*_*D*_*s* of approximately 1.2 mM for H3_9–18_K14ac (27), 500 μM for H3_3–17_K9acK14ac (28), and 900 μM for H3_9–19_K14ac (19) peptides.

### Structural basis of H3K14ac recognition by the CeSMARCA4 bromodomain

**Figure 3:**
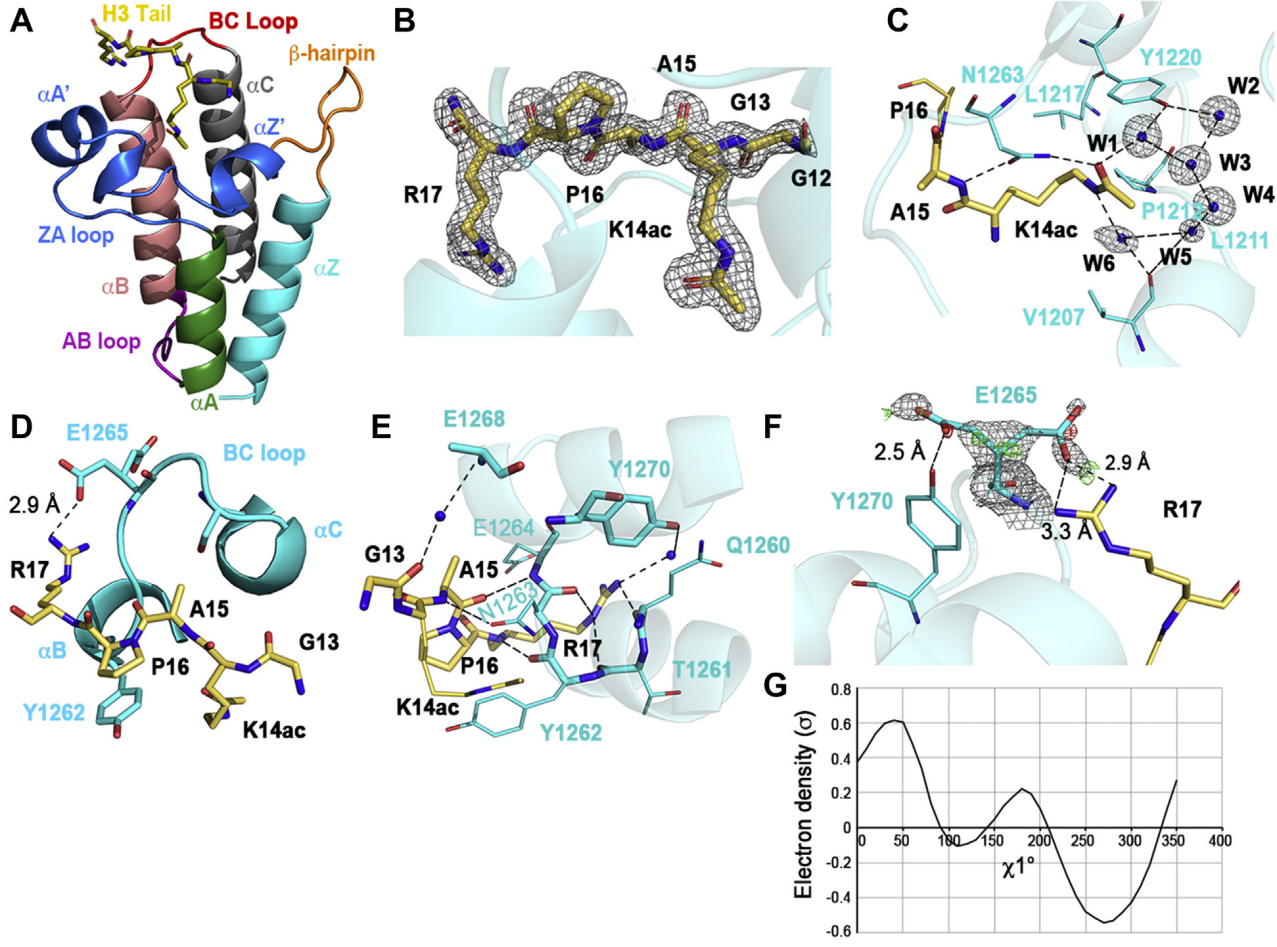
**Structure of the *Ce*SMARCA4 BD in complex with its modified histone H3K14ac target.***A*, structural features of the overall 1.29 Å *Ce*SMARCA4 bromodomain–H3_7–20_K14ac complex. The αZ, αA, αB, and αC helices are colored *cyan*, *green*, *dark salmon*, and *gray*, respectively. The ZA, AB, and BC interhelical loops are colored *blue*, *purple*, and *red*, respectively. The ZA loop includes two short helices, αA′ and αZ′, with a 3_10_ helical turn adjacent to αA’. The β-hairpin, a hallmark of family VIII bromodomains, is shown in *orange*. The modified histone H3 peptide is shown in *yellow*. *B*, residues G13-R17 can be unequivocally traced in the 2Fo-Fc electron density map, which is contoured at 1σ, while G12 can be partially traced. *C*, interactions with K14ac (*yellow*, *stick*) bound in the *Ce*SMARCA4 binding pocket (*cyan*). The conserved Asn1263 side chain forms a hydrogen bond with the acetyl carbonyl group of acetyl-lysine and also forms a hydrogen bond with the H3A15 amide nitrogen atom. A network of structural water molecules (*blue spheres* in 2Fo-Fc electron density map contoured at 1σ) mediate interactions between K14ac and the Val1207 carbonyl group, and Tyr1220 side chain. Hydrophobic interactions with the side chains of Val1207, Leu1211, Pro1212, and Leu1217 further stabilize K14ac in the bromodomain pocket. *D*, sidechain interactions of the H3 tail outside of the K14ac binding pocket. H3A15 binds in a small pocket adjacent to the BC loop. H3P16 contributes hydrogen stacking interactions, packing with the benzene ring of Tyr1262. And H3R17 forms a salt bridge with one conformation of the Glu1265 sidechain. *E*, backbone interactions by the bromodomain with the H3 tail outside of the K14ac binding pocket. Glu1264 and Tyr1262 form direct backbone contacts with the carbonyl of A15, and amide nitrogen of R17. The bromodomain Glu1268 amide nitrogen forms a water-mediated contact with the G13 carbonyl group. Three additional hydrogen bonds mediate R17 sidechain interactions: by Gln1260 and Thr1261 backbone contacts and a water-mediated contact with Tyr1270. *F*, the two conformations of Glu1265. The dominant rotamer (60% occupancy) forms a salt bridge with H3R17. The other rotamer (40% occupancy) forms a hydrogen bond with Tyr1275. The final 2mFo-DFc electron density (*grey*) is shown contoured at 0.8 σ. Very little +3σ (*green*) or −3σ (*red*) mFo-DFc density remain in the map. Maps were generated with Phenix (67) and displayed in Pymol (69). *G*, ringer plot of Glu1265 shows two chi1 peaks at ∼60° and ∼180° (40).

To understand how SMARCA4 interacts with modified histones, we determined the crystal structure of the *C. elegans* SMARCA4 BD (residues 1176–1296) in complex with an H3_7–20_K14ac modified peptide at 1.29 Å resolution (Table 1). The *Ce*SMARCA4 BD exhibits the canonical fold of a left-handed bundle of four α-helices (αZ, αA, αB, αC) linked by one long (ZA) and two short (AB and BC) interhelical loops (Fig. 3*A*). The ZA loop includes two short helices (αZ′ and αA′) and a 3_10_ helical turn preceding αA’. The four amphipathic α-helices are antiparallel and pack tightly against each other to define the hydrophobic cavity for acetyl-lysine recognition (38). Residues Tyr1196–Ile1204 fold into a β-hairpin structure characteristic of family VIII BD.

**Table 1:** Crystallographic statistics

Data statistics	
PDB Code	7LHY
Wavelength (Å)	1.0
Resolution range (Å)	48.8–1.29 (1.34–1.29)
Space group	P 41 21 2
Unit cell	69.06 69.06 55.32 90.00 90.00 90.00
Unique reflections	34,008 (3336)
Multiplicity	13.8 (12)
Completeness (%)	99.6 (100)
Mean I/sigma(I)	13.9 (2.1)
Wilson B-factor (Å^2^)	17.2
R-sym	0.075 (0.93)
Refinement statistics	
Resolution (Å)	26.97–1.29 (1.33–1.29)
R-work	0.1792 (0.2679)
R-free	0.1938 (0.2703)
Number nonhydrogen atoms	1022
Macromolecules	868
H3 peptide	39
Water	115
Protein residues	110
RMS(bonds)	0.007
RMS(angles)	0.935
Ramachandran favored (%)	100
Ramachandran outliers (%)	0
Average B-factor (Å^2^)	23.3
Macromolecules	22.3
H3 peptide	29.6
Solvent	31.3

The structure of the *Ce*SMARCA4 BD–H3_7–20_K14ac complex reveals the molecular histone–tail interactions of the SWI/SNF enzymatic core. Residues H3_13–17_ of the modified histone peptide could be unequivocally traced in the electron density map (Fig. 3*B*). The histone peptide lysine acetylamide binds within the central, largely hydrophobic cavity, as observed for other BDs (Fig. 3, *A* and *C*), including the recently reported family VIII, yeast RSC *Sc*Sth1 BD–H3K14ac complex (30, 38). The acetyl carbonyl group of H3K14ac forms hydrogen bonds with the conserved Asn1263 residue and a water molecule that bridges to the conserved Tyr1220 residue. These residues stabilize acetyl-lysine binding to BDs (38). Site-directed mutations of Tyr1220Ala or Asn1263Ala in the *Ce*SMARCA4 BD independently abolish binding to H3 N-terminal tails *in vitro*, as confirmed by ITC (Fig. 2, *A* and *B*). A network of six water molecules, one of which also mediates a hydrogen bond between K14 Nε and the Val1207 backbone carbonyl, are buried within the hydrophobic cleft. Four of the six waters are conserved across BD families. The hydroxyl of Tyr1287 in the *Sc*Sth1 structure forms a hydrogen bond with the H3K14ac amide; however, this interaction is absent in the *Ce*SMARCA4 structure.

Additional histone peptide residues also interact with *Ce*SMARCA4, either through the ZA- and BC-loop residues, or the αB- and αC-helices flanking the cleft. The H3G13 carbonyl is anchored to the αC-helix by a water-mediated hydrogen bond that bridges the main-chain amides of Glu1268 (Leu1545 in *Hs*SMARCA4/2) and Ile1269 (Fig. 3*E*). The H3G13 carbonyl interacts with Trp1338 in the *Sc*Sth1 structure. H3A15 and H3P16 are hydrophobic (Fig. 3*D*) and mutating them to hydrophilic residues reduced binding to the *Sc*Sth1 BD (30). The H3A15 sidechain packs adjacent to the BC loop, anchored by hydrogen bonds between the peptide main chain with the carbonyl δ_1_-oxygen of the conserved Asn1263 (Fig. 3*C*) and the backbone amide of Glu1264 (Leu1573 in *Hs*SMARCA4/2) (Fig. 3*E*). The H3P16 cyclic sidechain packs closely with the Tyr1262 benzene ring (Phe1571 in *Hs*SMARCA4/2) (Fig. 3*D*). While KacXXR occurs multiple times within H3 and H4 tails, the KacXPR sequence is unique to H3K14ac (39). H3P16 appears to provide binding specificity for *Ce*Smarca4 BD binding and positions H3R17 around the αB-helix to facilitate BD contacts (Fig. 3, *B* and *E*).

H3R17 contributes essential interactions for binding to H3K14ac—the H3R17A mutation abolishes binding in *Sc*Sth1 (30) and *Hs*BAZ2B (35). The backbone amide nitrogen of H3R17 forms a hydrogen bond with the Tyr1262 backbone carbonyl (Fig. 3*E*). The H3R17 sidechain is tethered to the BD αB-helix and BC loop *via* hydrogen bonds between the sidechain nitrogen atoms and the main-chain Gln1260, Thr1261, and Asn1263 carbonyl groups—all conserved in *Hs*SMARCA4/2 (Fig. 3*E*). Water-mediated hydrogen bonds stabilize the interaction between H3R17 and Tyr1270 on the αC-helix.

The guanidinium group of H3R17 adopts a single conformation in the *Ce*SMARCA4 structure, oriented toward Glu1265. The electron density for *Ce*SMARCA4 Glu1265 indicates the sidechain adopts multiple conformations (Fig. 3*F*). We modeled the population of Glu1265 rotamers with Ringer (40), which identified two chi-1 angles at approximately +60° and 180° (Fig. 3*G*). After refinement, the dominant rotamer (60% occupancy) forms a hydrogen bond with H3R17, while the secondary rotamer (40% occupancy) is oriented toward Tyr1270 (Fig. 3, *D* and *F*). The Glu1265 rotamer oriented toward Tyr1270 exhibits the same chi-1 angle as the dominant rotamer in the *Sc*Sth1, *Hs*SMARCA4 apo and PFI-3–bound BD structures (28, 30, 41). The H3R17 guanidinium group in the *Sc*Sth1 structure purportedly interacts with the π electrons of Phe1331 (*Sc*Sth1 numbering) (30). Phe1331 is not conserved in SMARCA4 (Thr1261 in *Ce* and *Hs*SMARCA4). In fact, H3R17 forms a hydrogen bond with different symmetry-mate residues in both the *Ce*SMARCA4 and *Sc*Sth1 structures, which appears to be an artifact (not shown). Overall, the conservation of Glu1265 argues that the H3R17-Glu1265 interaction is functionally important.

### Interpreting the specificity of H3K14ac recognition and its evolutionary conservation

**Figure 4:**
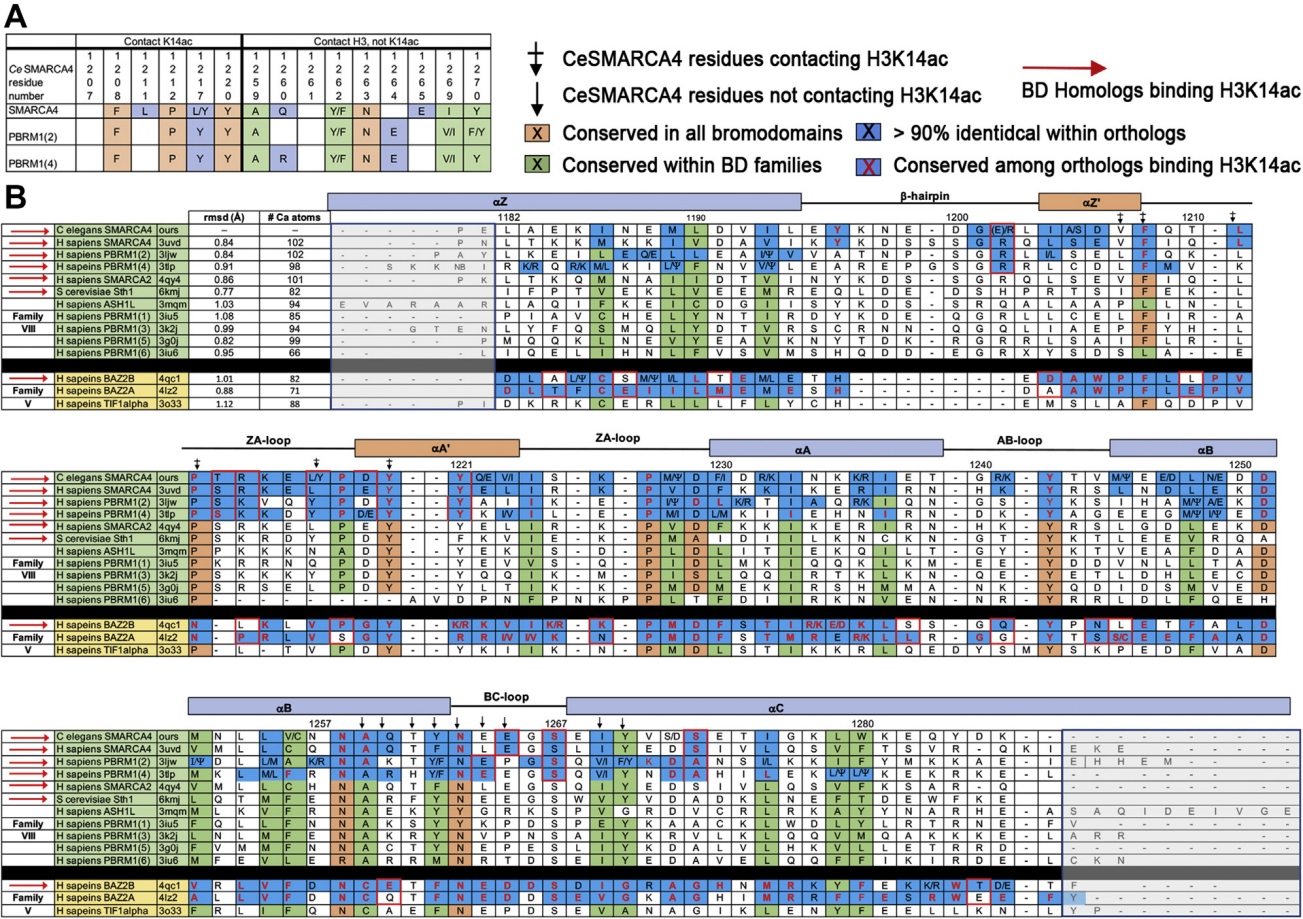
**Conserved residues in bromodomains that recognize H3K14ac.***A*, based on sequence conservation, the 15 residues in direct contact with the H3 peptide do not determine sequence specificity. Only the conserved residues are listed here—*blank boxes* are not conserved. The four *orange boxes* are conserved in all BDs, and the four *green boxes* are conserved within BD families (shaded as in (1)). *Boxes* colored *blue* are conserved for that paralog (SMARCA4 or PBRM1(2) or PBRM1(4)). The sidechains of Glu1264 or Glu1265 contact H3R17 and are the only paralog-specific residues that contact a sidechain of H3 outside of the K14ac binding pocket. *B*, the *Ce*SMARCA4 BD sequence is aligned with other *H sapiens* family VIII and family V sequences. The 15 residues contacting the H3K14ac peptide in *Ce*SMARCA4 are denoted as *black arrows*. *Boxes* are colored as in *A*. *Boxes* with *red outlines* identify conserved residues shared in family VIII BDs that bind the H3K14ac mark or residues that distinguish BAZ2A and BAZ2B BD orthologs. Secondary structure elements are marked along the *top*. *Numbers* above the sequence refer to the *Ce*SMARCA4 sequence. The alignment is based on structures (pdbid, third column) superimposed with the *Ce*SMARCA4 structure resulting in an overall root mean square deviation (rmsd, fourth column) of residues less than 2 Å apart after superposition (number of Cα atoms, fifth column). Structures with longer sequences outside of the aligned regions are included in the grey boxes at the N- and C-termini of the alignment. Ψ, hydrophobic residue; A_1_/A_2_, two similar residues occurring in the alignment.

Having defined the *Ce*SMARCA4 BD H3K14ac interactions, we next asked what the structure reveals about sequence-specific recognition of H3K14ac. The 15 *Ce*SMARCA4 BD residues contacting the H3 peptide are listed in Figure 4*A*. Contacting residues are defined as BD residues within 3 Å of the peptide or interacting with the peptide *via* water-mediated hydrogen bonds regardless of whether they are backbone or sidechain contacts. SMARCA4 is a member of the family VIII BDs (1). Surprisingly, eight of the *Ce*SMARCA4 residues contacting the H3K14ac peptide are conserved across all BDs, or among family VIII BDs (Fig. 4*A*).

**Figure 5:**
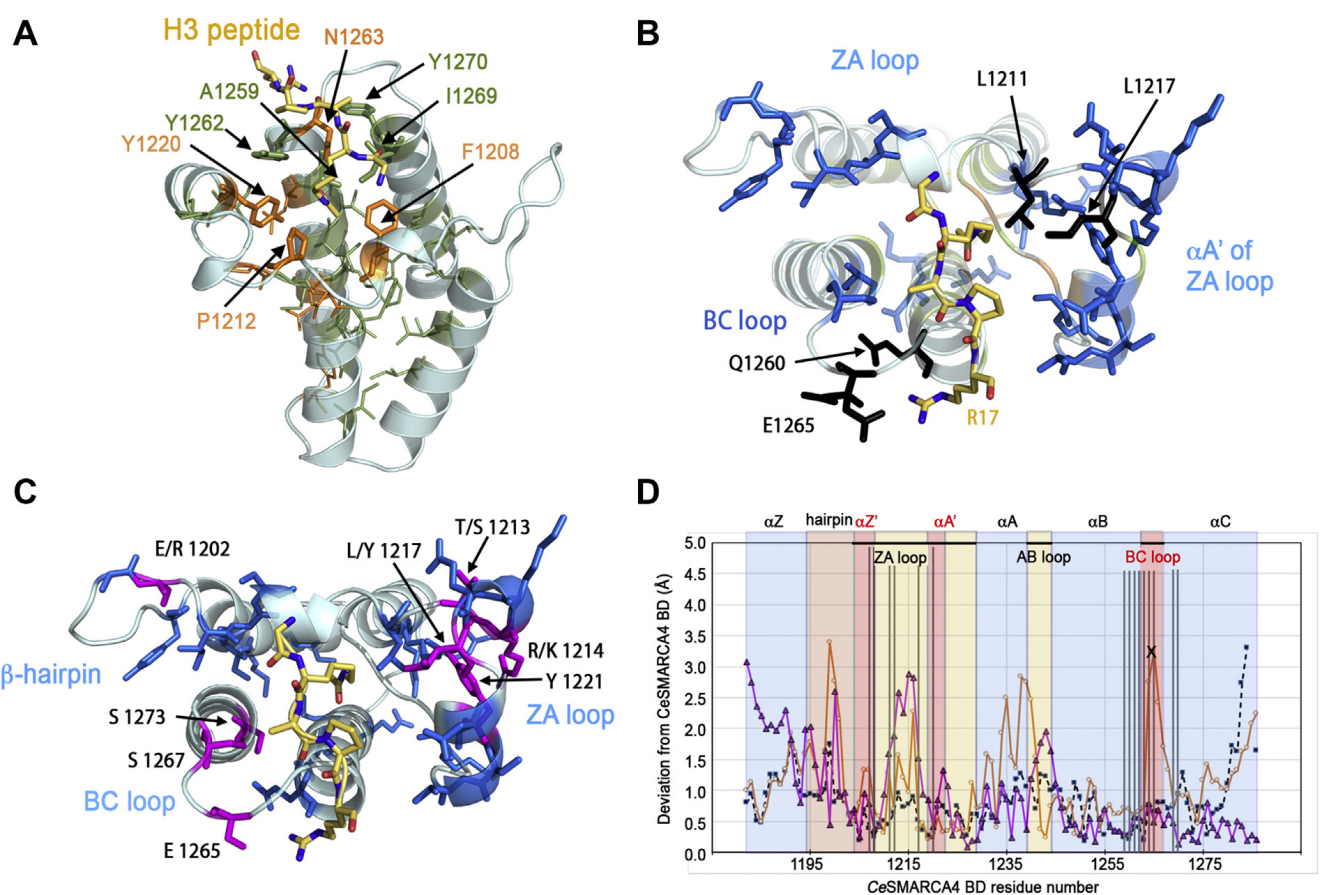
**Comparison between family VIII bromodomains.***A*, the 21 residues conserved in SMARCA4 BDs (*stick* representation) include residues conserved in all bromodomains (*orange*) and residues conserved in family VIII bromodomains (*green*). Most of the family VIII conserved residues are located in the protein core. Eight of the conserved residues (labeled) are among the 15 residues that contact the H3K14ac peptide: four are conserved in all BDs, and four are conserved in family VIII BDs. *B*, the 29 conserved SMARCA4 BD residues that may contribute to H3K14ac binding specificity. Residues colored *blue* (as in Fig. 4) are conserved in the *Ce*SMARCA4 alignment and not in family VIII BDs overall (Fig. 5*A*). Most of these residues are located in the loops or on the surface of the domain. The four *black residues* contact the H3K14ac_13–17_ peptide directly. Both conformations of Glu1265 are shown—one of which forms an electrostatic interaction with H3R17. *C*, conserved residues shared by family VIII BDs that bind the H3K14ac mark: SMARCA4, SMARCA2, PBRM1(2), and PBRM1(4) bromodomains (*colored magenta*, and highlighted with *red boxes* in the alignment in Fig. 4). These residues cluster on the ZA loop and the BC loop and the N-terminus of αC helix, including the acidic residue Glu1264 or Glu1265 that interact with H3K17R. Blue sidechains are similar in SMARCA4 orthologs, as in Figure 5*B*. Residues conserved in all family VIII BDs are excluded from this figure. *D*, H3K14ac-binding specificity results from positioning the contact residues. Each bromodomain structure was superimposed on the structure of CeSMARCA4-BD by minimizing the rmsd between Cα atoms, pruning residue pairs greater than 2 Å apart (overall rmsd reported in Fig. 4), and plotting the deviation per residue. The 15 *vertical lines* indicate contact residues, within 3 Å of the H3K14 peptide (or water-mediated contacts) in the *Ce*SMARCA4-BD structure (marked by *black arrows* in the alignment in Fig. 4). The deviations of the contact residues between *Hs*SMARCA4 and *Ce*SMACA4 (*dotted black line*, *square markers*), and between *Sc*Sth1 and *Ce*SMARCA4 (*purple line*, *triangle markers*) are low, 1.5 Å or less, indicating that these residues are positioned similarly in these three bromodomains. The deviations of the contact residues between *Hs*Ash1L and CeSMARCA4 (*orange line*, *open circle markers*) is large for residues in the BC-loop. In addition, the *Hs*Ash1L residue corresponding to residue 1265 (*Ce*SMARCA4 numbering) is an arginine instead of glutamate, preventing binding to H3R17 (marked with an “X”). The secondary structure elements are indicated along the *top* of the graph with *boxes* drawn below: α-helices colored *blue*, loops colored *beige* or *red*, the beta hairpin in *brown*. The H3 peptide contacts consist of regions around the ZA and BC loops.

Because both *Hs*SMARCA4 and *Ce*SMARCA4 BDs bind H3K14ac, we hypothesized that H3K14ac-binding specificity is evolutionarily conserved. We therefore investigated the extent of conservation among residues of SMARCA4 orthologs. First, we aligned 1000 full-length SMARCA4 sequences. From this alignment, we isolated the BDs, selected the 100 most diverse sequences, and scored the alignment for sequence conservation (see Experimental procedures). We found 50 conserved residues among all SMARCA4 BDs (Fig. 4*B* first row, blue boxes), 21 of which are conserved in all family VIII BDs (Fig. 5*A*). Except for Val1207, all residues in the H3K14ac binding pocket are conserved. Of the 29 SMARCA4-specific residues, about half are located on the BC and ZA loops surrounding the H3 peptide (Figs. 4*B* and 5*B*) while the other half are located on the BD surface. Four of the residues contacting the H3 peptide are SMARCA4-specific (Fig. 4*A*): Leu1211, Leu1217, the Gln1260 backbone contact with H3K14ac (Fig. 3, *C* and *E* and 5*B*), and the conserved Glu1265 residue, which forms a crucial salt bridge with H3R17 (Fig. 3*F*). Glu1265 is the only SMARCA4-specific sidechain in contact with an H3 peptide sidechain outside of the Kac-binding pocket.

We next asked if the SMARCA4-specific residues are also conserved in other family VIII BDs that bind H3K14ac. Three other Family VIII BDs are reported to selectivity bind H3K14ac: SMARCA2, *Sc*Sth1, and PBRM-1 (19, 30). PBRM-1 is an SWI/SNF accessory protein in the PBAF complex that contains six tandem N-terminal BDs (42). The second and fourth PBRM-1 BDs (BD(2) and BD(4)) bind H3K14ac (43, 44). Due to the low sequence homology outside of the central α A and αB helices (1), we aligned the family VIII BDs based on the superposition of their structures and added *Ce*SMARCA4 to the alignment of the human BDs (Fig. 4) (1). The 15 *Ce*SMARCA4 BD residues contacting the H3 peptide are marked with arrows above the columns on the sequence alignment (Fig. 4*B*). We found full conservation of these residues between SMARCA4 and *Hs*SMARCA2.

Aligning PBRM-1 sequences, we found PBRM-1 BD(2) and BD(4) each contain 43 conserved residues, 32 of which overlap (Fig. 4*B*, blue boxes). Very few SMARCA4-specific residues are conserved in PBRM-1 BD(2) and BD(4), and only eight are similar—Glu/Arg1202 (90% Arg), Ser/Thr1213, Arg/Lys1214, Leu/Tyr1217, Tyr1221, Glu1264/1265, Ser1267, Ser/Ala1273 (Fig. 5*C*). Residues Leu/Tyr1217 and Glu1264/1265 are in direct contact with the H3 peptide (Fig. 4*A*), the other conserved residues cluster around the ZA and BC loops. We conclude that each BD paralog encompasses a unique set of conserved residues, suggesting that various combinations of paralog-specific residues can contribute to H3 recognition.

Because the sequence of SMARCA4-BD residues in direct contact with the H3 peptide do not sufficiently explain binding specificity, we investigated whether the positioning of contact residues is critical for histone recognition. We superimposed each family VIII BD structure onto the *Ce*SMARCA4-BD structure and plotted the deviation for each residue (Fig. 5*D* and Fig. S2). We note that comparisons between apo and ligand-bound BD structures reveal very few conformational changes, suggesting that apo structures are sufficient to ascertain contact-residue positions (28, 30, 41). The overall root-mean-square deviation (rmsd) for each structure is indicated in Figure 4*B*. Our analysis reveals very low deviations (<1.5 Å) between *Hs*SMARCA4, *Ce*SMARCA4, and *Sc*Sth1 for residues in contact with the H3 peptide (Fig. 5*D*). In contrast, the deviation from *Ce*SMARCA4-BD is large in at least one of the contact residues for the ASH1L-BD, a methyltransferase that is unable to bind H3K14ac (1). The deviation of residue 1265 (CeSMARCA4 sequence) is over 3 Å, and the ASH1L Glu1265Arg substitution precludes H3R17 binding (Fig. 5*D*). Our analysis detected low deviations when comparing contact residues of HsPBRM-1 BD(2), BD(4), and BD(5) relative to CeSMARCA4 (Fig. S2*A*). The HsPBRM-1 BD(5) observation was interesting given that BD(5) cannot bind H3K14ac but reportedly assists PBRM-1–H3K14ac binding (43, 44). Glu1264 (not 1265) is conserved in PBRM-1 BD(2) and BD(4), and BD(4) features the Glu1264/Glu1265 acidic pair. It is likely that Glu1264 mediates the BD–H3R17 interaction in PBRM-1 BD(2) (Fig. 4 and Fig. S4*D*). *Hs*PBRM-1 BD(3) also shows low deviation at contact residues relative to *Ce*SMARCA4. BD(3) may be unable to bind H3K14ac because Val1264 and Pro1265 (*Ce*SMARCA4 numbering) substitute for acidic residues that contact H3R17 (Fig. 4*B* and Fig. S2*C*).

BAZ2B-BD binds H3K14ac but also recognizes poly-acetylated H3 tails (Fig. 1*D*). The family V BAZ2B-BD and the family VIII *Ce*SMARCA4-BD structures superimpose well (rmsd 1.0 Å for 82 of 105 Cα atoms). Although the histone peptide is shifted relative to the BDs, H3K14ac binds to BAZ2B and *Ce*SMARCA4 in the same conformation—the bound H3 peptides superimpose remarkably well (Cα atoms of H3G13–H3R17, rmsd 0.2 Å, Fig. S3). The deviation in the peptide-superimposed BAZ2B-BD and *Ce*SMARCA4-BD contact residues is largest for residues near K14ac (0.5–3 Å) and much smaller for residues near A15, P16, and R17 (1 Å or less, Fig. S2*D*). We found little sequence overlap when comparing the 29 SMARCA4-specific residues identified earlier with 50 conserved BAZ2B-specific residues (Fig. 4*B*). Five *Ce*SMARCA4 BD–H3K14ac contact residues (Val1207, Pro1212, Ala1259, Gln1260 and Tyr1270) are different in BAZ2B (Pro2084, Asn2920, Cys2136, Glu2137, and Gly2147, respectively). H3R17 binding in *Ce*SMARCA4 is mediated by Glu1265, whereas the BAZ2B BD features two conserved acidic residues (Glu2141, Asp2142) that interact with H3R17 (Figs. 4*B* and , S4*D*). We also compared the BDs of BAZ2B and BAZ2A—an NoRC complex BAZ2B homolog with an H4K16ac-binding BD (45). Glu2141 and Asp2142, which contact the H3K14ac_13–17_ peptide in BAZ2B, are conserved in BAZ2A (Fig. 4*B*). Superimposition of the BAZ2A and BAZ2B BD structures shows that the contact residues are in the same position (Fig. S2*E*). From these criteria, it is not possible to distinguish specificity for binding H3K14ac (KacAPR) by BAZ2B from H4K16ac (KacRHR) by BAZ2A. Both histone sequences include arginine as the fourth residue. In contrast to BAZ2A, superimposing the TRIM24/TIF1α BD (family V) with the BAZ2B BD clearly indicates that TRIM24/TIF1α does not bind H3K14ac due to significantly different positioning of peptide-binding residues (Fig. S2*E*).

With the exception of the role of Glu1264-Glu1265 in H3R17 recognition, our comparative data shows that the BD residues in direct contact with histone tail sequences do not, by themselves, account for H3K14ac specificity. Rather, binding specificity appears to be dependent on positioning the contact-residues for a given histone sequence. Conserved SMARCA4-BD loop residues likely contribute to positioning the H3K14ac contacts (Fig. 5*B*). The overall conservation of BD residues for each paralog suggests that multiple residue combinations can prime similar contacting positions to facilitate H3K14ac recognition.

### *In vivo* role of the CeSMARCA4 BD in SWI/SNF function

To investigate the *in vivo* biological function of SMARCA4 BD–H3K14ac binding, we engineered an Asn1263Ala putative loss-of-function mutation into the *C. elegans* SMARCA4 protein, encoded by the *swsn-4* gene, using CRISPR-Cas9 genome editing (46). We hypothesized that disruption of this key residue, which is critical for binding to H3K14ac-modified H3 tails *in vitro* (35, 37) would also disrupt the BD’s ability to bind H3K14ac *in vivo*. To our surprise, we found that *Ce*SMARCA4 Asn1263Ala mutant worms exhibited no obvious defects in viability, growth, or development compared with wild-type animals when synchronously grown under standard conditions at 20 °C or 25 °C (data not shown). We subsequently generated in-frame deletion mutants targeting the BC loop–H3R17 interactions and similarly observed that disruption of the Glu1264-Glu1265 acidic pair had only modest effects on worm viability.

Given the incongruity between our *in vitro* and *in vivo* results, particularly when considering the high conservation of the *Ce*SMARCA4 BD and histones across all metazoans, we contemplated whether (1) other accessory SWI/SNF subunits may function redundantly *in vivo*, and (2) our SMARCA4 BD mutants may exhibit higher penetrance when introduced into a sensitized genetic background. For instance, synthetic lethality has been reported in *swsn-4* and *swsn-1/SMARCC2* double mutants (47) (the *swsn-4* mutations in that study were in the ATPase domain, rather than the BD), and low penetrance has been individually observed with weak alleles of *swsn-4* and *swsn-1* in cell- and tissue-specific contexts (48).

**Figure 6:**
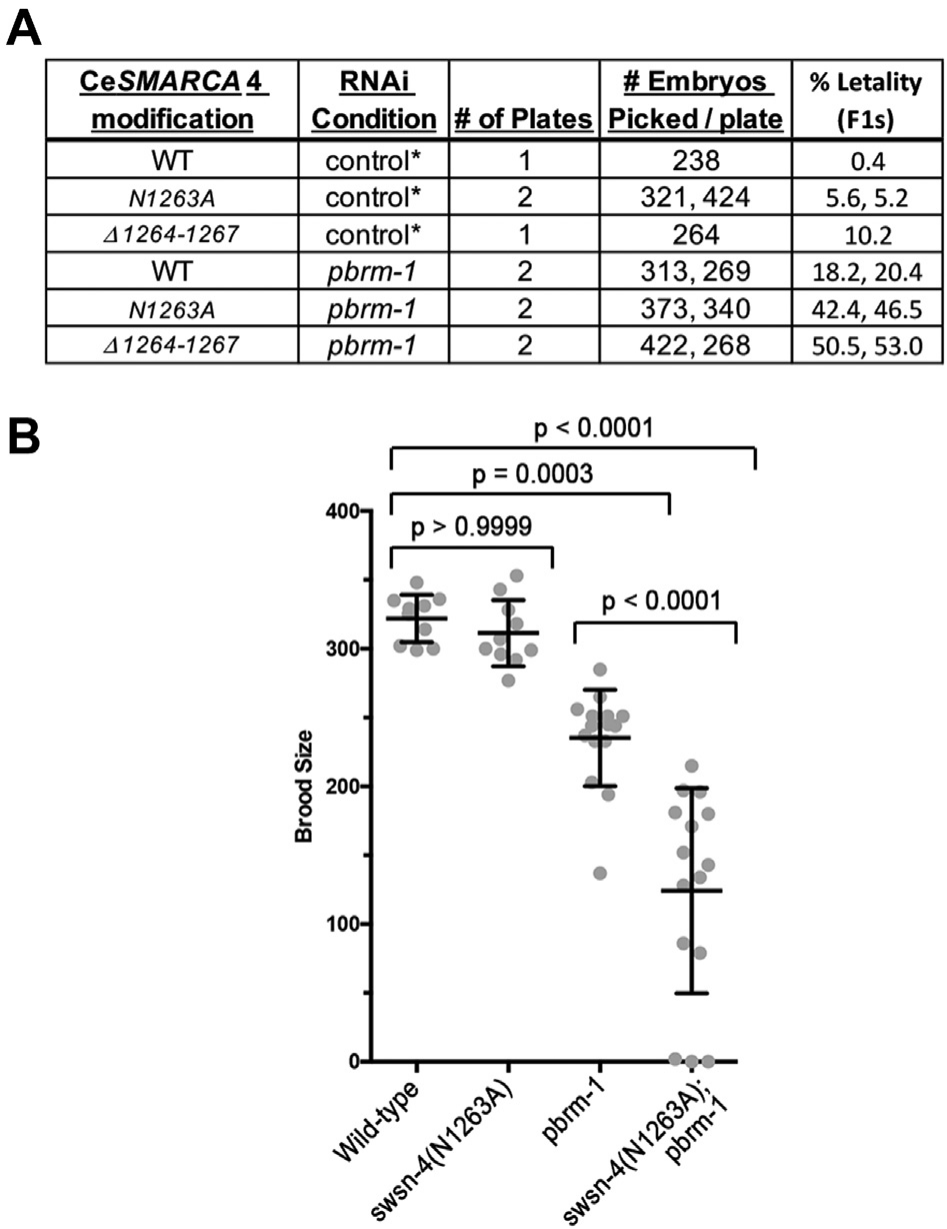
**CeSMARCA4 and PBRM-1 bromodomains function together to regulate *C elegans* development.***A*, RNAi knockdown of *pbrm-1* resulted in synergistic embryonic lethality with the *swsn-4(Asn1263Ala)* and *swsn-4(Δ1264–1267)* mutations. Embryos from P0 animals fed *pbrm-1* or control RNAi were scored as viable if they developed to the L3 stage at 20 °C. *B*, brood sizes of individual *swsn-4* and *pbrm-1* mutants and the double mutant. Individual brood sizes are plotted for each genotype and the error bars indicate the standard deviation of brood (n = 12–17). The mean of the *swsn-4(Asn1263Ala)* brood size was not significantly different from the wild-type worms. The reduced brood size in the *pbrm-1* mutant, and the *swsn-4(Asn1263Ala); pbrm-1* double mutant, was significant (one-way ANOVA).

To further interrogate *Ce*SMARCA4 function *in vivo*, we knocked down candidate BD-containing proteins in wildtype and *swsn-4*(*rhd138[Asn1263Ala]*) mutants by RNAi. The candidate target genes included *swsn-1* and *swsn-5* (the other core SWI/SNF subunits), *swsn-7* (a PBAF ARID domain-containing subunit), *swsn-9* (ortholog of human BRD9 and BRD7 proteins), *pbrm-1* (which encodes PBRM-1/BAF180 and comprises six tandem N-terminal BDs), and *let-526* (a PBAF ARID domain-containing subunit) (49). Surprisingly, we detected enhanced levels of embryonic and larval lethality in *swsn-4*(*Asn1263Ala*) animals treated with *pbrm-1* RNAi (Fig. 6*A*). The combined lethality was synergistic, suggesting that SWSN-4 and PBRM-1 may have overlapping roles. We also tested *pbrm-1* RNAi in the *swsn-4*(Δ*1264–1267*) mutants bearing the BC-loop deletions and observed similar results to the *swsn-4*(*Asn1263Ala*) mutants. These data clearly demonstrate that *pbrm-1* knockdown by RNAi enhances embryonic lethality of *swsn-4* mutant animals.

To corroborate the genetic interaction observed in our RNAi experiments, we generated the *swsn-4*(*rhd138[Asn1263Ala]*); *pbrm-1*(*tm415*) double mutant, which we subsequently refer to as *swsn-4*(Asn*1263Ala*); *pbrm-1*. The *pbrm-1*(*tm415*) deletion mutation is a presumptive null allele (42). The brood size of the *swsn-4*(*Asn1263Ala*) BD mutant did not differ significantly from wild-type worms, as corroborated by one-way ANOVA analyses (Fig. 6*B*). In contrast, the mean brood size for the *pbrm-1* mutant was decreased by 27% relative to wild-type worms (*p*-value = 0.0003). Importantly, the brood size of the *swsn-4; pbrm-1* double mutant was decreased by 61% relative to wild-type worms (*p*-value < 0.0001). These results demonstrate a synergistic genetic interaction between the two loss-of-function alleles.

Collectively, our results show that disruption of the *Ce*SMARCA4 BD function *via* mutation of Asn1263, which is critical for H3K14ac binding *in vitro*, or deletion of the acidic BC-loop residues (Glu1264-Glu1265) involved in H3R17 binding selectivity, does not impact *C. elegans* viability. However, when the *pbrm-1* gene was inactivated in the *swsn-4*(*Asn1263Ala*) and *swsn-4*(*Δ1264–1267*) mutant backgrounds, the double mutants exhibited enhanced embryonic lethality and reduced brood sizes, revealing that the SMARCA4 BD indeed is playing a significant role *in vivo*, but likely works in an overlapping manner with other accessary members of the SWI/SNF complex.

## Discussion

Targeting SWI/SNF complexes to chromatin is key to regulating DNA access required for transcriptional control (50), DNA damage repair (51, 52), and replication (53). Three distinct SWI/SNF chromatin remodeling complexes have been identified from mammalian cells, each containing a core SMARCA4/BRG1 or SMARCA2/BRM catalytic subunit (54). Mutations in SWI/SNF proteins are common to many cancers, acting as tumor-suppressors or oncogenes in different contexts (55). Efforts to design small-molecule inhibitors to disrupt SMARCA4 histone interactions have focused on binding to the bromodomain Kac binding pocket (41). The current paper addresses the binding specificity and function of the conserved BD of SMARCA4/BRG1 in regulating acetylated histone tails. Notably, our investigation into the *in vivo* role of the *Ce*SMARCA4 BD marks the first report showing that this reader domain is required for SWI/SNF function in any organism.

SMARCA4 is the only SWI/SNF core subunit in *C elegans*, which lacks a SMARCA2 homolog. The *Ce*SMARCA4 BD specifically binds mono-acetylated H3K14 tails *in vitro*, like *Hs*SMARCA4. We initially found that BD mutations that abolish acetyl-lysine binding *in vitro* have little to no effect on *C. elegans* viability. However, combining BD mutants with the loss of PBRM-1, an accessory component of the SWI/SNF PBAF complex (48), revealed a critical role for this domain. As PBRM1 has two BDs that bind to H3K14ac, we speculate that these additional BDs function redundantly with the *Ce*SMARCA4 BD in large, multi-subunit complexes such as SWI/SNF BAF and PBAF (48). Indeed, redundancies between multiple members of a chromatin complex have been seen by others (56, 57). However, we cannot rule out the possibility that *Ce*SMARCA4 and PBRM-1 act in parallel, and another functional feature of PBRM-1 loss leads to the combinatorial phenotype. Future studies will be needed to define the basis of this phenotype and redundancy, although these studies nonetheless define a critical function of the *Ce*SMARCA4 BD. It would be intriguing to explore whether this same relationship exists in human SWI/SNF.

We report the first SMARCA4-BD structure bound to an H3_7–20_K14ac modified peptide. The position of H3K14ac contacts in our *Ce*SMARCA4 structure superimpose closely with the corresponding residues in the apo *Hs*SMARCA4 BD structure, suggesting a lock-and-key binding mode. Of note, many of the contact residues that compose the 15 sidechain, backbone and water-mediated contacts by the *Ce*SMARCA4-BD with H3_13–17_K14ac are shared among family VIII BD, including BDs with different histone-binding profiles (1). It is therefore critical to compare related BD structures and sequences to interpret binding specificity.

We propose that BD sequences that contribute to binding specificity are evolutionarily conserved among SMARCA4 orthologs. SMARCA4-BD-specific residues were identified by searching the database with full-length *Ce*SMARCA4 and aligning the BDs from the resulting sequences. The alignment included 29 SMARCA4-specific residues, excluding residues conserved among family VIII BDs (Fig. 4) (1). Glu1265 is the only conserved residue that directly contacts the H3K14ac peptide outside of the K14ac binding pocket. In the *Ce*SMARCA4 BD structure, H3R17 is oriented toward Glu1265 about 60% of the time. In contrast, H3R17 in the *S. cerevisiae* Sth1 (*Sc*Sth1) BD structure interacts with a nonconserved Phe (30). The conservation of Glu1265 suggests that the Glu1265-H3R17 interaction is essential. Glu1265 may neutralize the H3R17 charge *in vivo* in the context of SMARCA4 complexes that would otherwise bury the H3R17 charge. Previous studies have indirectly interrogated the acidic residue interaction with the H3 tail in the *Hs*BAZ2B bromodomain *in vitro via* R17A mutation or deletion of H3R17 (35, 58). However, those approaches do not specifically test the contribution of the acidic pair residues, as they also abolish critical direct hydrogen bonds between the R17 sidechain and the BD backbone observed in our structure.

The other conserved SMARCA4-specific residues are dispersed along the ZA and BC loops and on the protein surface (Fig. 5*B*). Our hypothesis is that the conserved loop residues contribute to binding specificity by positioning the contact residues to interact with H3_13–17_K14ac. In support of this hypothesis, family VIII BD structures that bind the H3K14ac mark position the peptide-contact residues similarly to *Ce*SMARCA4-BD (Fig. 5*D* and Fig. S2). This includes SMARCA4, SMARCA2, the second and fourth BDs in Polybromo-1 (PBRM-1(BD2) and PBRM-1(BD4)), as well as the fifth BD (PBRM-1(BD5)), which has been shown to assist in binding to H3K14ac (28, 43, 44). Family VIII BDs that do not bind H3K14ac either alter the position of the contact residues or lack an acidic residue to bind H3R17.

To determine if the same residues are conserved in all family VIII BDs that bind H3K14ac, we aligned orthologs of the other BDs that recognize this mark. We found that a different set of residues are conserved in each paralog, including different residues along the ZA and BC loops. Glu1264 (using *Ce*SMARCA4 numbering) is conserved in PBRM-1(BD2) and PBRM-1(BD4) instead of Glu1265, and likely interacts with H3R17 (Fig. 4). Despite the variation of conserved residues among these paralogs, the position of the contact residues in family VIII BDs coincides with the contact residues of CeSMARCA4-BD, as indicated above (Fig. 5*D* and Fig. S2). We conclude that specificity for binding the H3K14ac mark results from a combination of BD geometry, positioning the contact residues, and an acidic residue to bind H3R17. Future studies will test this model by swapping conserved residues between BD paralogs.

The current study focuses on the question of binding specificity. In characterizing the *Ce*SMARCA4 BC, we measured the K_d_ of the *Ce*SMARCA4 BD for H3K14ac peptides by ITC as low micromolar affinity, like other high-affinity BD-peptide interactions, including the ScSth1 BD from the RSC complex (1, 30). This is consistent with the essential role of the SMARCA4 BD. The *Hs*SMARCA4 BD was reported to bind an H3K14ac peptide with a K_d_ of low millimolar affinity as measured by NMR perturbation, a thousand-fold lower than the *Ce*SMARCA4 BD (19, 27, 28). This discrepancy is surprising given the similar positioning of the peptide contacting residues (Fig. 5*D*). The lower affinity of the *Hs*SMARCA4 BC may result from the different measurement techniques. In addition, different length H3 peptides are utilized in these studies ranging from H3_8–18_K14ac to H3_1–25_K14ac (30, 59). Similar discrepancies with NMR measurements have been reported elsewhere, for example the K_d_ for polybromo BRD2 was reported as ∼500 μM by NMR (60), and single-digit μM values by fluorescence anisotropy (59, 61). A future study is required that systematically compares binding affinity of BDs binding H3K14ac.

We extended the positional and evolutionary conservation analysis to BAZ2B, a family V BD that binds H3K14ac (35, 36). The residues conserved in the BAZ2B BD differ from residues conserved in the SMARCA4 BD, consistent with the diversity of sequences able to configure the required geometry to bind H3K14ac. Five H3_13–17_ K14ac contact residues differ between *Hs*BAZ2B and CeSMARCA4, and the binding site is shifted relative to the family VIII bromodomains. BAZ2B includes acidic residues, Asp1264 and Glu1265 to interact with H3R17, at the equivalent position to *Ce*SMARCA4 Glu1265. The comparison between family V and family VIII BDs suggests histone recognition of H3K14ac is a product of convergent evolution among distinct bromodomain families.

Our study has implications for the development of small-molecule BD inhibitors. Functional redundancy as identified in SWI/SNF BDs may partly explain the acquired resistance and off-target effects broadly seen in recent literature analyzing BD inhibition *in vivo* (see, *e.g.*, (11, 12, 13, 62)). Many BD inhibitors have targeted binding to the H3Kac binding pocket (7, 63). Our comparison shows that the H3K14 binding pocket of family VIII BDs is highly conserved (Fig. 4*A*). SMARCA4 residues contacting the H3 peptide outside of the binding pocket are positioned to contact the H3K14ac mark, but other family VIII BDs have similar profiles, particularly SMARCA2, PBRM1(2), and PBRM1(4), which bind H3K14ac. A SMARCA4-specific inhibitor could also target additional contact residues, particularly Glu1265 that interacts with H3R17. Such an inhibitor might bind the other BDs that target the H3K14ac mark. Our structural and sequence analysis indicates that binding specificity for histone marks is dispersed over the ZA and BC loops. Inhibition of SMARCA4 BD specifically might exploit these unique conserved sequences outside of the peptide-binding site, for example, Q1260, which is conserved in SMARCA4 but not PBRM1(2) or PBRM1(4). Targeted CRISPR-Cas9 BD mutations provides an endogenous control to corroborate small-molecule inhibitor experiments.

## Conclusions

The histone code is based on differential binding specificity of epigenetic readers such as the BD. BD binding specificity for H3K14ac is evolutionarily conserved among SMARCA4 orthologs. From alignments of family VIII BD sequences, we show that binding specificity by SMARCA4-BD does not just depend on the residues in direct contact with the H3K14ac but includes residues in the ZA and BC loops. We demonstrate for the first time that the *Ce*SMARCA4 BD is required for SWI/SNF *in vivo*. Our data suggests that the function of the CeSMARCA4 BD may be redundant with PBRM-1 in the pBAF complex. Functional redundancy is likely a common feature of epigenetic readers, an important consideration for future drug design efforts.

## Experimental procedures

### Cloning

The *C. elegans* full-length *swsn-4* (the human *SMARCA4* ortholog) sequence was PCR-amplified from wild-type *C. elegans* Bristol N2 cDNA. The PCR product was used as template to subclone the *swsn-4* bromodomain sequence encoding residues D1179–N1289 into a pGEX vector with an N-terminal GST tag. PCR amplification was performed using Q5 high-fidelity DNA polymerase (M0491, New England BioLabs). The *Ce*SMARCA4 BD PCR products were phosphorylated with T4 polynucleotide kinase, treated with *Dpn*I, and ligated into the pGEX vector using T4 DNA ligase (New England BioLabs). The ligation mixture was used for transformation into *E. coli* DH5α competent cells. Recombinant colonies were confirmed by Sanger sequencing. The Asp1179–Asn1289 construct was used for protein characterization on histone-peptide microarrays and ITC.

The *swsn-4* BD construct used for crystallization (residues Lys1176–Glu1296) was PCR-amplified from cDNA, subcloned into a pGEX-6P-1 vector featuring an N-terminal GST tag and a human rhinovirus (HRV) 3C protease cleavage site, and sequenced as indicated above. The crystallization construct contains an Phe1287Ser point mutation.

### Protein expression and purification

For recombinant protein expression, the *Ce*SMARCA4 GST–BD construct was transformed into *E. coli* BL21 (DE3) cells and grown at 37 °C in LB-Ampicillin supplemented broth from overnight cultures to an O.D. 600 of 0.6 to 0.7. Protein expression was induced with 1 mM isopropyl β-D-1-thiogalactopyranoside (IPTG), followed by overnight protein expression at 22 °C. Cultures were harvested by centrifugation and bacterial pellets were resuspended in lysis buffer (1× Phosphate Buffer Saline (PBS), 5 mM Methionine, pH 7.3). A high-pressure homogenizer or sonicator was used for lysis at 4 °C. Lysates were cleared by centrifugation, applied to Glutathione Sepharose 4B affinity chromatography resin (GE Healthcare) for batch purification at 4 °C, and washed with lysis buffer. The resin was then loaded on a column and washed extensively with wash buffer (10 mM HEPES, 100 mM NaCl, 2.5% glycerol, pH 7.3) at 4 °C.

For characterization on histone-peptide microarrays and ITC, the GST-BD protein was eluted from the column using elution buffer (10 mM HEPES, 100 mM NaCl, 2.5% glycerol, 10 mM reduced glutathione, pH 7.3) and dialyzed overnight against wash buffer at 4 °C. The eluted protein was analyzed by SDS-PAGE (12.5% NEXT Gel, VWR).

For crystallization, the GST-BD protein was treated overnight with PreScission Protease at 4 °C to cleave the N-terminal GST tag and loaded into a column packed with Q Sepharose high-performance, anion-exchange resin (GE Healthcare) for purification by ion-exchange chromatography. The protein was eluted in a linear salt gradient (0.1–1 M NaCl) and dialyzed against wash buffer at 4 °C. Size-exclusion chromatography on a HiPrep 16/60 Sephacryl S-200 HR (GE Healthcare) column was performed as a polishing purification step. The untagged *Ce*SMARCA4 BD peak fractions were dialyzed against crystal-preparation buffer (10 mM HEPES, 100 mM NaCl, pH 7.3) at 4 °C. Protein purity was analyzed by SDS-PAGE before concentrating the protein to 21 mg/ml for crystallization with an Amicon centrifugal concentrator (3 MWCO).

### Crystallization

Concentrated *Ce*SMARCA4 BD was incubated in 2:1 M excess histone H3_7–20_K14ac peptide ligand at 4 °C. Crystallization screens of the complex were set up by sitting-drop vapor diffusion at 4 °C using a polyethylene glycol (PEG) smear-based BCS custom-made screen (64). A well-ordered crystal with prism morphology grew in 35% PEG 400, 550 MME, 600, and 1000. The crystal was mounted on a cryoloop, flash frozen in reservoir solution with liquid nitrogen, and shipped to the Advanced Photon Source, Argonne National Laboratory SER-CAT 22-ID beamline.

### Data collection, processing, and structure determination

A complete diffraction dataset for the *Ce*SMARCA4 BD-H3_7–20_K14ac crystal was collected and processed to 1.29 Å resolution. The data were integrated and scaled with HKL2000 (65). Initial phases were determined by molecular replacement using Phaser (66) as implemented in Phenix (67). The initial search model for the *Ce*SMARCA4 BD–H3_7–20_K14ac crystal structure was derived from the *Hs*SMARCA4 apo bromodomain (PDB 2GRC) (28). Automated rebuilding of the model was accomplished with Autobuild in Phenix, followed by iterative rounds of Phenix refinement and manual rebuilding in COOT (68). The *Ce*SMARCA4 BD-H3_7–20_K14ac complex crystallized in space group P4_1_2_1_2 with a monomer in the asymmetric unit. Figures were generated using PyMol (69).

### Isothermal titration calorimetry

All calorimetric measurements were performed on a MicroCal Auto-iTC200 (Malvern Panalytical) instrument at 293 or 298 K in 10 mM HEPES, 100 mM NaCl, 2.5% glycerol, pH 7.3. Purified *Ce*SMARCA4 BD protein solution (50–100 μM) in the calorimetric cell was titrated against histone H3_7–20_K14ac peptide solution (500–1000 μM) in the syringe. The data was fit into a single-site binding model with the MicroCal Origin software. The experiments’ first data points were excluded from the analysis.

#### Histone peptide microarrays

Peptide synthesis, array preparation, and effector protein analysis were performed as described previously (34, 70). The arrays were scanned (Typhoon Trio+ Imager, GE Healthcare) and protein–peptide interactions were quantified by fluorescence (ImageQuant array software, GE Healthcare). The signal from each of the spots for each peptide was averaged, values were normalized to the highest-calculated value across all peptides, and subsequently plotted on a scale from 0 to 1.

### Bromodomain alignments

Due to low sequence conservation among BD proteins, *Ce*SMARCA4 BD orthologs were identified in BLASTp by searching with the full-length SMARCA4 protein (71). One thousand SMARCA4 sequences were identified by searching the nonredundant database. The alignment included diverse species from *H. sapiens* to *S. cerevisiae* (available upon request). The BD sequences were then isolated from the full-length SMARCA4 sequences (with Bali-Phy (72)), aligned (with Seaview (73)), paired down to the 100 most diverse sequences (with Bali-Phy), and scored for sequence conservation using the JS Divergence scoring method (https://compbio.cs.princeton.edu/conservation/index.html) (74), which scores conservation by the BLOSUM62 matrix with a moving window of three amino acids. Residues with a score of 2.2 or higher were considered similar, unless alignments contained 10% or more nonsynonymous residues.

One-thousand PBRM-1 orthologs were identified in BLASTp by searching with full-length *Hs*PBRM-1. Ortholog sequences from various species comprising at least 25% sequence homology were selected. PBRM-1(BD2) and PBRM-1(BD4) were extracted and realigned as indicated above. The 116 (BD2) and 97 (BD4) most diverse sequences were analyzed for conservation.

We identified conserved residues in BAZ2B by searching the nonredundant database in BLASTp for sequences similar to full-length *Hs*BAZ2B (71). We searched for 5,000 sequences to increase the diversity of the alignment. The range of species represented was smaller than the Family VIII alignments, restricted to chordates including amphibians, sea turtles and fish. *C elegans* encodes a single BAZ2 homolog of unknown specificity instead of the two paralogs: BAZ2B and BAZ2A. The BAZ2B bromodomains were spliced out of the aligned sequences, realigned, and the list was pruned to 132 of the most diverse sequences. BAZ2A sequences were selected from the same 132 species as the final BAZ2B alignment. One-hundred and two BAZ2A sequences were found in the NCBI proteins database, all annotated as BAZ2A (71).

### *C. elegans* strains

Strains were cultured on *E. coli* OP50 using standard methods (75). The following *C. elegans* strains were used in this study: N2 Bristol (wild-type): GE24 *pha-1(e2123) III*, DLS479 *swsn-4(rhd138[Asn1263Ala]) IV*, DLS481 *swsn-4(rhd140[Δ1264–1267]) IV*, HS1222 *pbrm-1(tm415) I*, DLS489 *pbrm-1(tm415) I; swsn-4(rhd138[Asn1263Ala]) IV*.

### CRISPR-Cas9 genome editing constructs

The protospacer adjacent motif (PAM) targeting the *swsn-4* locus encoding the *Ce*SMARCA4 bromodomain Asn1263 residue was selected manually by searching for NGG sequences in either strand nearby the target insertion site. We used the MIT CRISPR design tool to analyze and check the specificity of the sgRNA target site ( http://crispr.mit.edu). The sgRNA was introduced into pJW1285 by Q5 site-directed mutagenesis and microinjected with the Asn1263Ala repair oligo (5′GCTTGTGAACAATGCTCAAACATACGCCGAGG AGGGCAGTGAGATTTATGTTAGCTCTGA3′) into gonads of young adult *pha-1(e2123)* mutant animals, as described previously (46). Injected animals were grown at 25 °C and viable F1 animals carrying the repaired *pha-1(e2123)* allele, which may be positive for the co-CRISPR event, were transferred to individual plates. The genomic DNA containing the desired lesions in the *swsn-4* gene was amplified by PCR and subjected to Sanger sequencing.

### RNAi and lethality assays

Treatment of N2, DLS479, and DLS481 animals with RNAi was performed as previously described (76). Briefly, gravid adults were grown on 10 cm plates and eggs were isolated by bleaching. Animals were synchronized in M9 media overnight and dropped on RNAi plates containing approximately 20x concentrated bacteria. P0 animals were grown at 20 °C for 3 days before embryos were transferred to new RNAi plates containing approximately 1× concentrated bacteria and grown at 20 °C. Animals that grew to the L3 stage were considered viable. All plates were monitored for at least 7 days.

### Brood-size measurements

Well-fed wild-type N2, DLS479, HS1222, and DLS489 strains were grown on OP50 for a least two generations. Brood-size measurements were performed as previously described (77). The largest and smallest brood-size values were excluded from the analysis for each strain.

## Data availability

Structure factors and coordinates for the crystal structure of CeSMARCA4 BD bound to the H3_7–20_K14ac peptide have been deposited in the Protein Data Bank (https://www.rcsb.org) with accession code 7LHY. Sequence alignments of SMARCA4, PBRM-1, BAZ2B, and BAZ2A BD are available upon request. All other data are included within the document.

## Supporting information

This article contains supporting information (1, 30, 35, 37, 41, 78).

## Conflict of interest

B. D. S. is a cofounder of EpiCypher, KK has an equity interest in EpiCypher.
