# Supplementary material for: Binding specificity and function of the SWI/SNF subunit SMARCA4 bromodomain interaction with acetylated histone H3K14
Source: J Biol Chem. 2021 Aug 30;297(4):101145. doi: 10.1016/j.jbc.2021.101145 (PMC8506967; doi:10.1016/j.jbc.2021.101145)
Supplement: Supplemental Figures S1–S4 [file mmc1.pdf]

Binding specificity and function of the SWI/SNF subunit SMARCA4 bromodomain interaction with acetylated histone H3K14

Paul Enríquez, Krzysztof Krajewski, Brian D. Strahl, Scott B. Rothbart, Robert H. Dowen, and Robert B. Rose

SUPPORTING INFORMATION FIGURES

Figure S1: Superposition of bromodomains that bind the H3K14ac mark.
Figure S2: H3K14ac-binding specificity results from positioning contact residues
Figure S3: Differences in binding of H3K14ac by *Hs*BAZ2B vs *Ce*SMARCA4
Figure S4: Conserved residues in BAZ2B

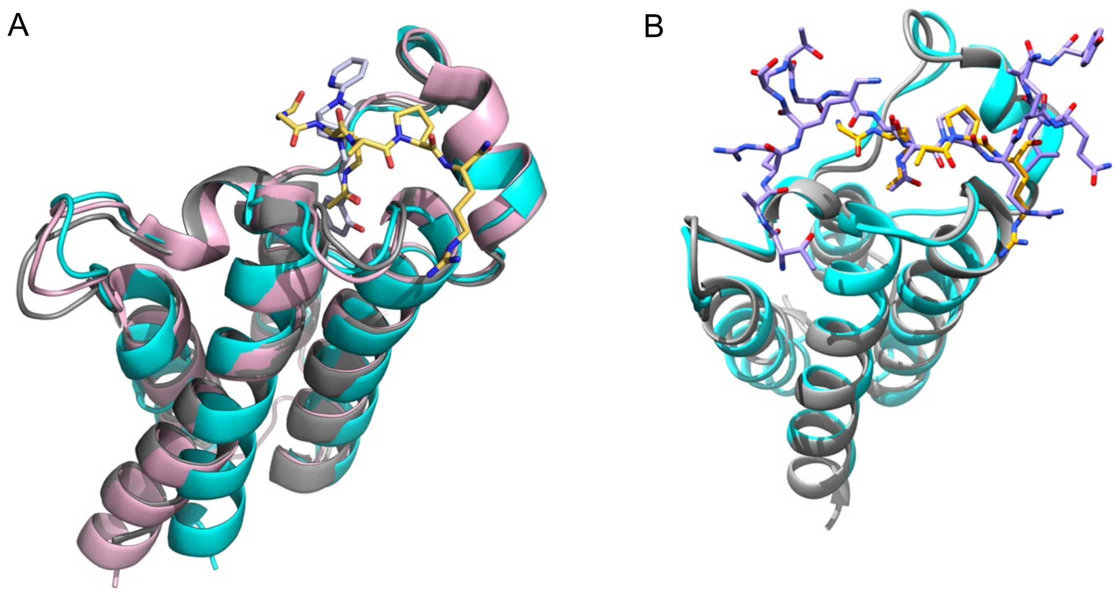

Figure S1: Superposition of bromodomains that bind the H3K14ac mark.
Superimposition of the *Ce*SMARCA4–H3$_{7\text{-}20}$K14ac (cyan) with A, *Hs*SMARCA4 bromodomain (grey cartoon) bound to the inhibitor PFI-3) [5DKD] (unpublished) (grey ligand) and B, the *S cerevaseia* Sth1 bromodomain (grey cartoon) bound to H3$_{7\text{-}20}$K14ac (purple sticks) [6KMJ] (30).

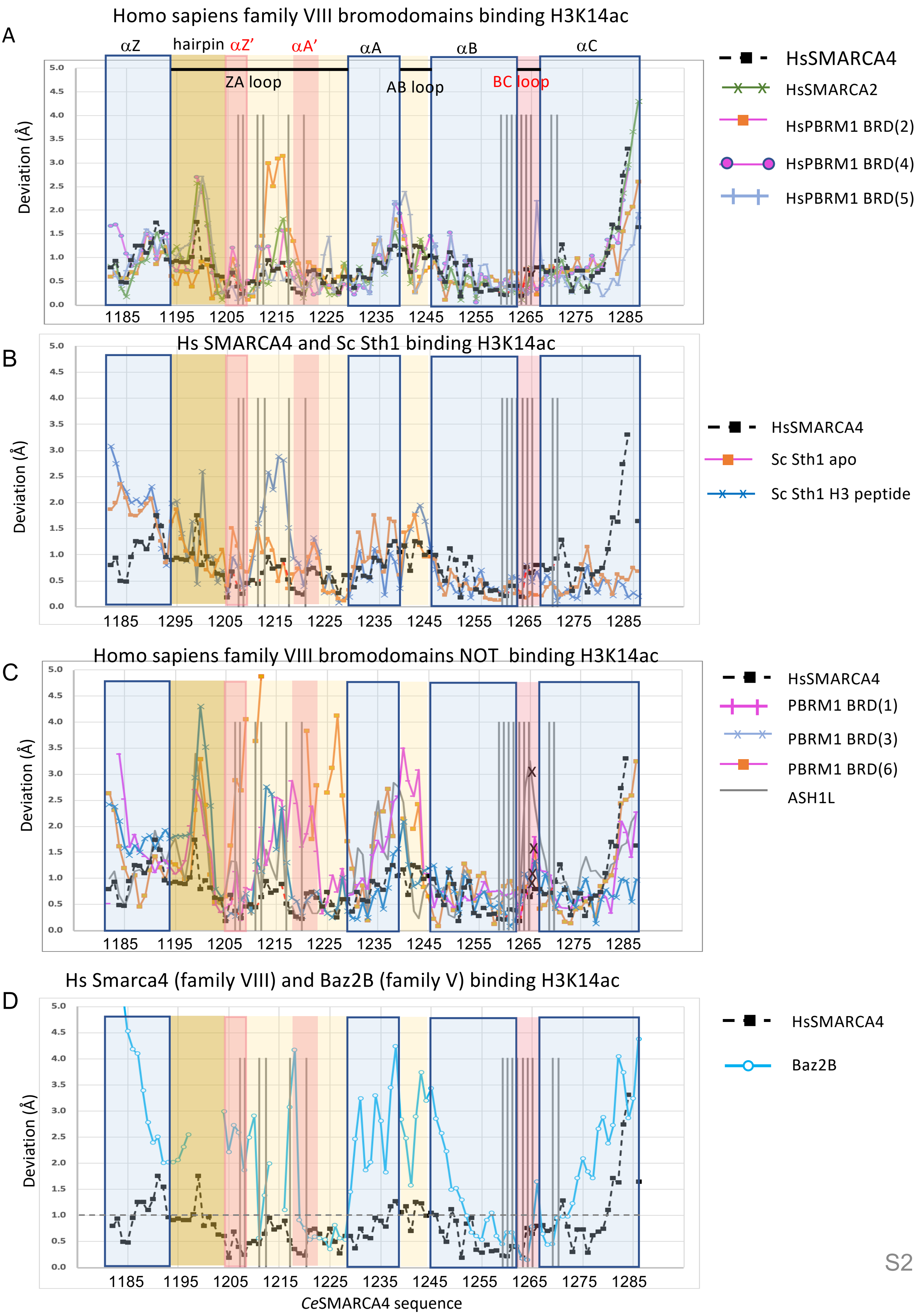
A
Homo sapiens family VIII bromodomains binding H3K14ac
αZ
hairpin
αZ’
αA’
αA
αB
αC
ZA loop
AB loop
BC loop
Deviation (Å)
HsSMARCA4
HsSMARCA2
HsPBRM1 BRD(2)
HsPBRM1 BRD(4)
HsPBRM1 BRD(5)
B
Hs SMARCA4 and Sc Sth1 binding H3K14ac
HsSMARCA4
Sc Sth1 apo
Sc Sth1 H3 peptide
C
Homo sapiens family VIII bromodomains NOT binding H3K14ac
HsSMARCA4
PBRM1 BRD(1)
PBRM1 BRD(3)
PBRM1 BRD(6)
ASH1L
D
Hs Smarca4 (family VIII) and Baz2B (family V) binding H3K14ac
HsSMARCA4
Baz2B
0.0
0.5
1.0
1.5
2.0
2.5
3.0
3.5
4.0
4.5
5.0
1185
1195
1295
1205
1215
1225
1235
1245
1255
1265
1275
1285
CeSMARCA4 sequence

S2

E

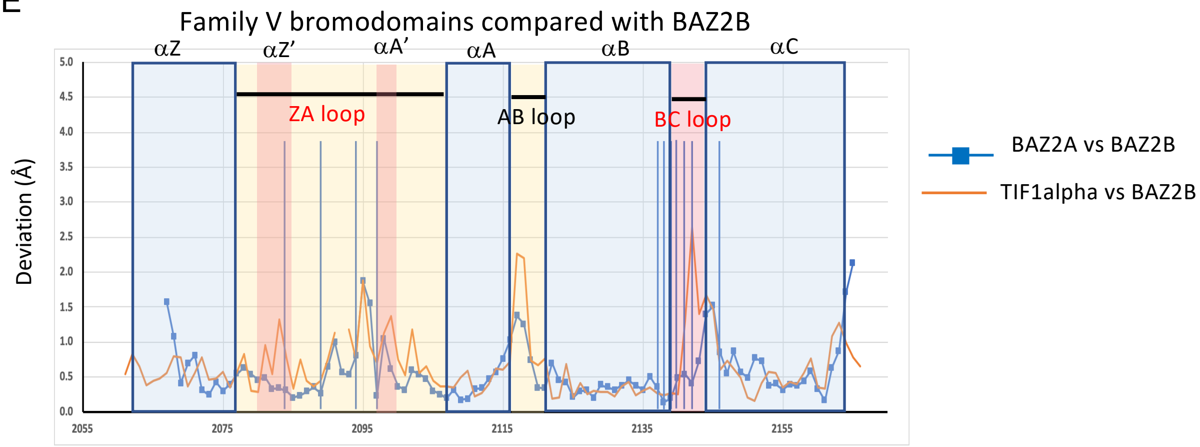

Figure S2. **H3K14ac-binding specificity results from positioning contact residue**s

Structural comparison of bromodomains show positioning of residues contacting the H3Kac peptide, as described for Figure 5D. (A-C) The deviation between each bromodomain and the *Ce*SMARCA4 bromodomain are displayed per residue. Vertical lines indicate the 15 CeSMARCA4 residues in direct contact with the H3K14ac peptide or forming a water-mediated contact. The difference between *Hs*SMARCA4 (1) and *Ce*SMACA4 are displayed on each of the graphs for comparison (dotted black line with black square markers). The secondary structure elements are indicated along the top of the graphs with boxes drawn below: α–helices colored blue, loops colored beige or red, the family VIII-specific beta hairpin in brown. Residues in the ZA loop contact K14ac in the central cavity, and residue around the BC loop interact with other H3 residues.

A, Family VIII bromodomains that bind the H3K14ac mark orient the contact residues similarly (rmsd < 1.5 Å).
B, The contacting residues in apo (pdbid 6kmb) and H3K14ac-bound (pdbid 6kmj) *Sc* Sth1 are also oriented like CeSMARCA4-BD. The conformational differences in the ZA loop do not affect H3K14ac13-17 binding (30).
C, Family VIII bromodomains that do not bind H3K14ac show large deviations at some contacting residues. PBRM1(3) is an exception – the contact residues are positioned like in CeSMARCA4-BD . The 'x' marks indicate that none of these bromodomains include Asp or Glu at 1264 or 1265, precluding interaction with H3R17.
D, Comparing *Hs*Smarca4 from family VIII with *Hs*BAZ2B from family V which also binds H3K14ac. The H3K14ac peptides were superimposed between BAZ2B and *Ce*SMARCA4 in order to compare the positions of the contacting residues. For all other superpositions the BDs were superimposed. The peptide in BAZ2B binds in a different orientation than in SMARCA4.
E, Family V bromodomains are compared with *Hs*BAZ2B . BAZ2A contacting residues are similar to BAZ2B (37), but not to TIF1alpha (80). The secondary structure elements and H3 peptide contacting residues differ slightly from family VIII due to the shorter ZA loop and absence of the hairpin.

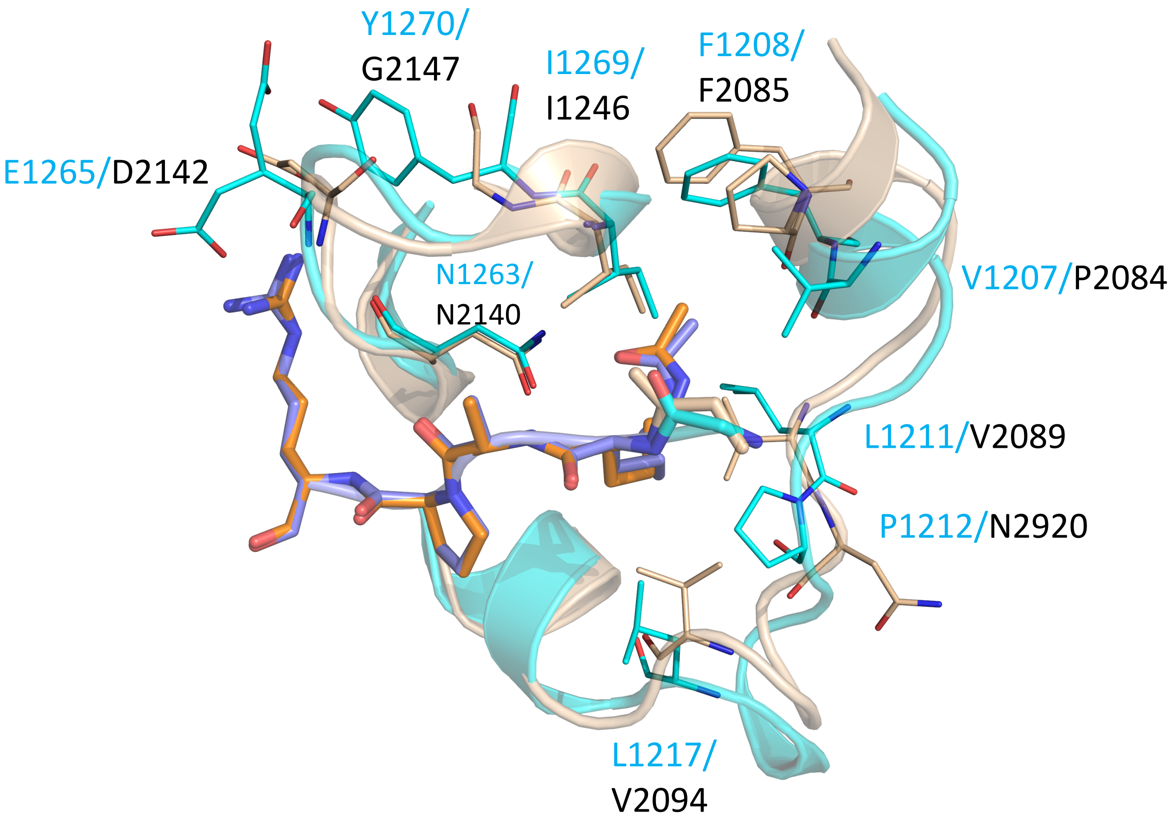

Figure S3. Differences in binding of H3K14ac by HsBAZ2B (wheat, purple H3 peptide) (35) **vs CeSMARCA4** (cyan). The H3K14ac peptides were superimposed. Five of the H3K14ac contact residues differ in BAZ2B: Val1207/Pro2084, Pro1212/Asn2920, Ala1259/Cys2136, Gln1260/Glu2137, Tyr1270/Gly2147 (numbering: *Ce*SMARAC4/*Hs*BAZ2B). BAZ2B includes three conserved acidic residues that could contact H3R17 like *Ce*SMARCA4 Glu1265: (Glu2141, Asp2142, Asp2143).

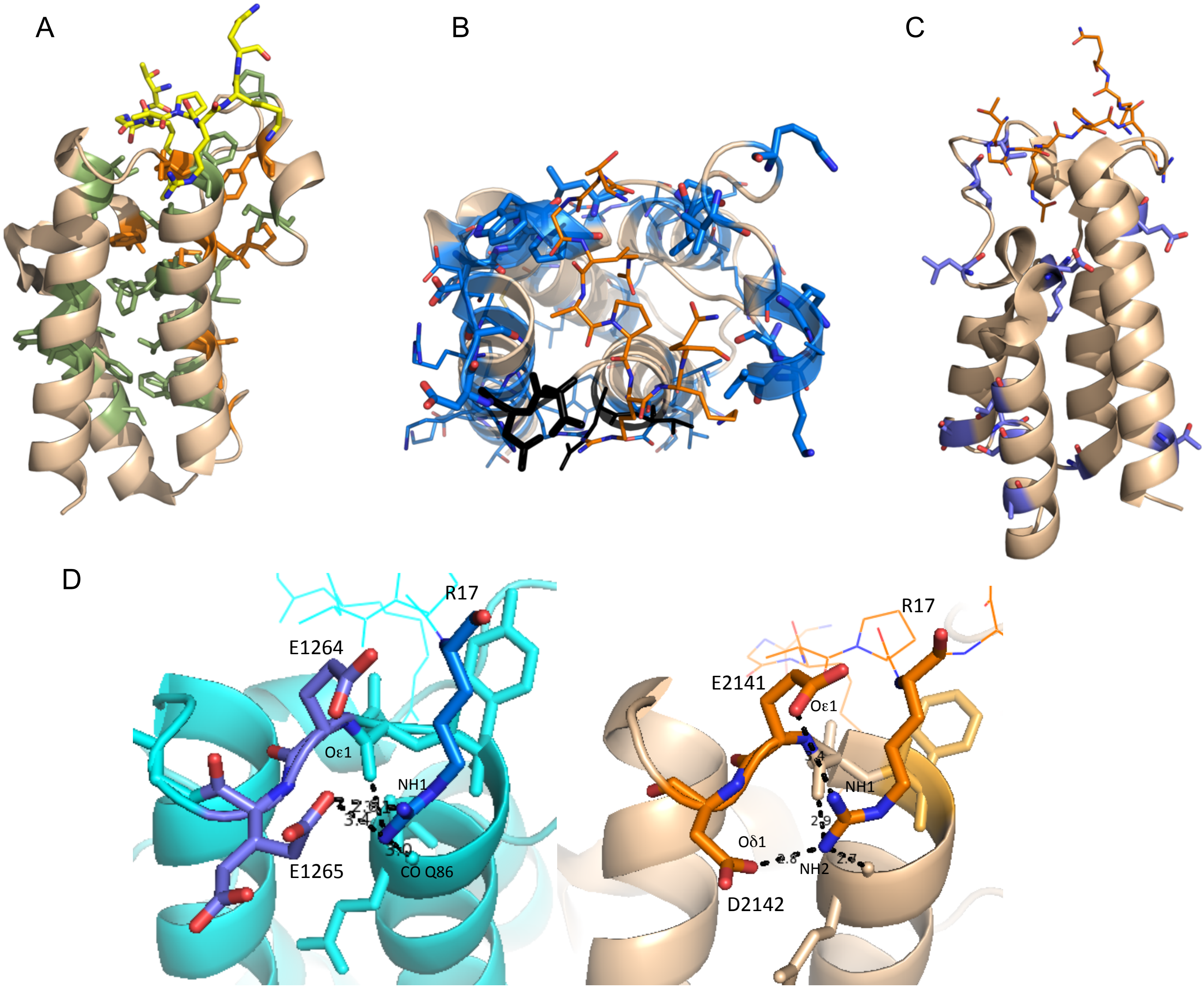

SMARCA4 H3R17 **BAZ2B H3R17**

Figure S4. Conserved residues in BAZ2B

A, 29 Residues in the BAZ2B alignment that are conserved in all bromodomains (orange) or in Family V bromodomains (green). As in the SMARCA4 paralog, most of these residues are hydrophobic, composing the core of the domain (See Fig. 5A). The colors match the residue colors in Fig. 4, and (1). These include 3 of the 7 residues that contact the H3 peptide outside of the Kac binding pocket.

B, 50 Conserved residues in the BAZ2B alignment that differ from the residues conserved in all Family V bromodomains, shown in Figure S4A. Most of these residues are located in the loops (sticks) or on the surface of the domain (lines). These residue are shaded blue, as in Fig. 4. The black residues contact the H3K14ac$_{13\text{-}17}$K14ac peptide (orange, sticks) directly.

C, Residues conserved in BAZ2B but not in BAZ2A. The structure is BAZ2B (pdb 4qc1) with the H3Kac14 peptide bound (orange sticks) (41). These residues are denoted in Fig 4 with red boxes around Family V residues. These are all surface residues, suggesting differences in protein-protein interactions.

D, Interactions between H3R17 and the BC loop acidic pair. Left: Interactions between H3R17 (blue) and the BC loop Glu1265 (purple) in *Ce*SMARCA4–H3$_{7\text{-}20}$K14ac. Right: Interactions between H3R17 and the acidic pair, Glu2141 and Asp242, in *Hs*BAZ2B–H3$_{11\text{-}19}$K14ac bromodomain complexes (pdb 4qc1) (37).
